# Supplementary material for: Development of a breast cancer risk assessment and primary prevention pathway for women aged 30–39 years: Views of UK primary care providers on the role of primary care
Source: PLoS One. 2024 Sep 13;19(9):e0308638. doi: 10.1371/journal.pone.0308638 (PMC11398678; doi:10.1371/journal.pone.0308638)
Supplement: S1 File — (DOCX) [file pone.0308638.s001.docx]

**S1 File.** Focus group and interview topic guide

**QUESTIONS**

1. As a researcher who does not work in primary care, I am not familiar with what happens in practice. It would help my understanding if you could you give me an example of when you’ve had a woman in this age group of 30-39 years present with a concern about their breast cancer risk or about breast health and talk through what happened and what you did.

*Probes:* what are/were your reasons for doing that? How did you feel about the interaction? How concerned do you think colleagues are about potential litigation?

*Prompts:* (if not answered) is this something you would deal with in your role, does this fit in your role, what would you do

**Breast cancer becomes more common in women in their 30s and is the most common cause of death in women aged 35-50. Before the age of 50 years, at least 65% of women who develop breast cancer do not have a family history and are not currently identified as being at increased risk.**

**Currently, there is no defined systematic mechanism to identify this group of women. The introduction of breast cancer risk assessment for women aged 30-39 years would allow women to find out their risk of developing breast cancer in the future. Women identified as being at increased risk could then be offered earlier breast screening as well as methods to reduce breast cancer risk. One potential approach is for breast cancer risk assessment and some aspects of risk management to be conducted in primary care.**

1. What are your immediate thoughts and reactions to offering women the opportunity to find out their breast cancer risk estimate from the age of 30 years?

*Probe:* what are your reasons for feeling that way?

1. What are your immediate thoughts and reactions to primary care identifying and inviting women to a breast cancer risk assessment?

*Prompts:* should it be primary care’s responsibility, how would this be organised – remit of breast screening programme or centrally organised, specialised service

1. What are your immediate thoughts and reactions to primary care involvement in breast cancer risk assessment and management?

*Prompts:* how acceptable do you think it would be, what would acceptability depend upon

*Probe:* what are your reasons for feeling that way?

**Risk of developing breast cancer is best calculated with a combination of three measures:**

**The following information known to impact breast cancer risk would need to be collected:**

- **Height and weight**
- **Family history of breast and ovarian cancer**
- **Age at first period**
- **Age of first pregnancy**
- **Oral contraceptive history**
- **Alcohol consumption**

1. What do you think about primary care collecting information from women about the list of breast cancer risk factors?

*Prompts:* family history (how many affected first or second-degree relatives and age of onset), hormonal factors, alcohol consumption

1. How would you feel about primary care performing this task?
2. Who would you envisage performing this task at your practice?

*Prompts:* would any additional staffing be required – primary care or risk prediction specialist, would it be appropriate to ask women to enter their own data

*Probe:* what are your reasons for feeling that way?

1. What are the key issues/difficulties/barriers to performing this task?
2. What would be required to perform this task successfully?

*Prompts:* any training/support needs, adaptations to infrastructure, guidelines

**One model of how breast cancer risk assessment could work in primary care is the development of a risk assessment tool similar to QRisk. For example, scores for mammographic density and genetic risk could be fed into the tool and a risk score generated once someone in primary care has entered family history, hormonal and lifestyle factors. Primary care would then be responsible for communicating the risk score and making a management plan.**

1. What do you think about primary care co-ordinating the process of breast cancer risk assessment in this way?
2. How would you feel about taking on this role?

*Prompt:* would it be appropriate/make sense to be involved in saliva sample collection too

1. Who would you envisage taking on this role at your practice?

*Prompt:* would any additional staffing be required – primary care or risk prediction specialist, who would input the information if women filled in form themselves

*Probe:* what are your reasons for feeling that way?

1. What are the key issues/difficulties/barriers to taking on this role?

*Prompts:* would another model work better

1. What would be required to take on this role successfully?

*Prompts:* any training/support needs, design considerations for risk assessment tool (e.g. integration with GP records), guidelines

**The output of the tool would also include recommendations for management of increased risk. Two strategies that have proven benefit in reducing breast cancer risk are:**

1. **Maintaining a healthy weight through diet and exercise and limiting alcohol intake**
2. **Taking risk-reducing medication such as tamoxifen**

**These risk management options would need to be discussed and offered to women identified at increased risk.**

1. What do you think about primary care providing lifestyle advice about reducing breast cancer risk?
2. How confident do you feel about your practice providing lifestyle advice?

*Prompts:* signposting to services – appropriate for breast cancer risk, thoughts on specific (for breast cancer risk) vs generic services (e.g. weight management)

*Probe:* what are your reasons for feeling that way?

1. Do you think there’s anything different about providing lifestyle advice with respect to breast cancer risk in comparison with other diseases?
2. Who would you envisage taking on this role at your practice?

*Prompt:* would any additional staffing be required – primary care or risk prediction specialist

*Probe:* what are your reasons for feeling that way?

1. What are the key issues/difficulties/barriers to taking on this role?
2. What would be required to take on this role successfully?

*Prompts:* any training/support needs, adaptations to infrastructure, guidelines

1. What do you think about primary care discussing and prescribing risk-reducing medication such as tamoxifen?
2. How confident do you feel about your practice discussing and prescribing risk-reducing medication?

*Probe:* what are your reasons for feeling that way?

1. Who would you envisage taking on this role at your practice?

*Prompt:* would any additional staffing be required – primary care or risk prediction specialist

*Probe:* what are your reasons for feeling that way?

1. What are the key issues/difficulties/barriers to taking on this role?
2. What would be required to take on this role successfully?

*Prompts:* any training/support needs, adaptations to infrastructure, guidelines

1. Do you think setting up a pathway for breast cancer risk assessment and management activities in primary care is a worthwhile idea? (if not, why not?)

*Prompts:* should it be primary care’s responsibility, would incentives like QOF points help, could/should it be integrated into existing health checks (e.g. cervical screening)

1. What other issues would be important to consider when setting up a pathway for breast cancer risk assessment and management activities in primary care?

**Finishing comments**

Thanks for your time today. We do really appreciate it.

- Is there anything else you want to add?
- Is there anything you thought you would talk about today which you haven’t had a chance to say and want to mention?
- What do you think is the most important message coming from this group today?
