# Supplementary material for: Development of a breast cancer risk assessment and primary prevention pathway for women aged 30–39 years: Views of UK primary care providers on the role of primary care
Source: PLoS One. 2024 Sep 13;19(9):e0308638. doi: 10.1371/journal.pone.0308638 (PMC11398678; doi:10.1371/journal.pone.0308638)
Supplement: S2 File — (DOCX) [file pone.0308638.s002.docx]

**S2 File.** Pre-reading material

Breast cancer becomes more common in women in their 30s and is the most common cause of death in women aged 35-50. Before the age of 50 years, at least 65% of women who develop breast cancer do not have a family history and are not currently identified as being at increased risk.

Currently, there is no defined systematic mechanism to identify this group of women. The introduction of breast cancer risk assessment for women aged 30-39 years would allow women to find out their risk of developing breast cancer in the future. Women identified as being at increased risk could then be offered earlier breast screening as well as methods to reduce breast cancer risk. One potential approach is for breast cancer risk assessment and some aspects of risk management to be conducted in primary care.

Risk of developing breast cancer is best calculated with a combination of three measures:

The following information known to impact breast cancer risk would need to be collected:

- Height and weight
- Family history of breast and ovarian cancer
- Age at first period
- Age of first pregnancy
- Oral contraceptive history
- Alcohol consumption

One model of how breast cancer risk assessment could work in primary care is the development of a risk assessment tool similar to QRisk. For example, scores for mammographic density and genetic risk could be fed into the tool and a risk score generated once someone in primary care has entered family history, hormonal and lifestyle factors. Primary care would then be responsible for communicating the risk score and making a management plan.

The output of the tool would also include recommendations for management of increased risk. Two strategies that have proven benefit in reducing breast cancer risk are:

1. Maintaining a healthy weight through diet and exercise and limiting alcohol intake
2. Taking risk-reducing medication such as tamoxifen

These risk management options would need to be discussed and offered to women identified at increased risk.
