# Supplementary material for: Development of a breast cancer risk assessment and primary prevention pathway for women aged 30–39 years: Views of UK primary care providers on the role of primary care
Source: PLoS One. 2024 Sep 13;19(9):e0308638. doi: 10.1371/journal.pone.0308638 (PMC11398678; doi:10.1371/journal.pone.0308638)
Supplement: Attachment — Submitted filename: SH Qual paper response to reviewers comments 15july2024.docx [file pone.0308638.s003.docx]

| **Editor comments** | **Author response** | **Page and line number** |
| --- | --- | --- |
| After careful consideration, we feel that it has merit but does not fully meet PLOS ONE’s publication criteria as it currently stands. Therefore, we invite you to submit a revised version of the manuscript that addresses the points raised during the review process.  The paper tackles an interesting topic that is useful to a global audience. The language and grammar still need editing and proof-reading. Also kindly explain the thematic analysis process as pointed out by the reviewer and explain the implications of your work to the global readership. | We thank the Editor for the opportunity to revise our manuscript based on the thorough comments from the reviewers.  We have responded to all comments and indicated where we have changed the text within the manuscript below (and using track changes in the manuscript). We hope you will now be able to accept our manuscript submission for publication in PLOS One but we are happy to respond to any further questions and comments you may have. | N/A |

**Responses to editor and reviewers’ comments**

| **Reviewer 1 comments** | **Author response** | **Page and line number** |
| --- | --- | --- |
| The paper presents important qualitative data on the views of primary care providers with respect to pro-active identification of women at higher risk of cancer under the current screening age. The paper is well written, with a clear rationale, well justified methods and appropriate results and conclusions. The results provide useful and comprehensive views from healthcare professionals that will be very informative in the field as risk assessment is implemented. The limitation section also provides interesting reflection on the strengths and limitations of the study design that will be useful for future researchers. | We are pleased that the reviewer considered the manuscript to be well written and a useful contribution to the body of existing knowledge. We would like to thank the reviewer for their suggestion to improve the paper. We have addressed their comment, with changes to the text tracked in the accompanying manuscript. | N/A |
| Line 74 mentions a change in NICE guidelines – could you briefly mention exactly what the change entailed? | Thank you for this suggestion. We have added detail about what the change entailed. The text now reads:  However, since June 2022, the proactive identification of women at increased risk of breast cancer within primary care has been facilitated by a change to National Institute for Health and Care Excellence (NICE) guidance **whereby the recommendation to identify women only when they present with concerns was withdrawn.** | Pg 4, lines 80-81 |

| **Reviewer 2 comments** | **Author response** | **Page and line number** |
| --- | --- | --- |
| Strengths  1. Relevance and Importance: The topic is highly relevant, considering the increasing incidence of pre-menopausal breast cancer and the potential benefits of early risk assessment and prevention.  2. Methodology: The use of focus groups and individual interviews allows for a comprehensive understanding of primary care providers' views.  3. Clear Presentation of Findings: The manuscript is well-structured and well-written. | We are pleased that the reviewer considered the manuscript to be well written and a useful contribution to the body of existing knowledge. We would like to thank the reviewer for providing constructive comments to improve the paper. We have addressed each of their comments, with changes to the text tracked in the accompanying manuscript. | N/A |
| Generalizability: The study is limited to primary care providers in Greater Manchester and Cambridgeshire and Peterborough. It would be beneficial to discuss the potential for generalizing these findings to other regions or countries.  Recommendation: Add detailed information on how the findings might apply to primary care settings outside the study locations. | Thank you for this suggestion. We have added a paragraph discussing transferability of our findings to the strengths and limitations section. Note we have used the term transferability instead of generalisability to reflect the terminology adopted in qualitative research:  **Participants were recruited from two regions with diversity in their patient populations in terms of socioeconomic status and ethnicity. However, they are both in England which limits the transferability of the findings as countries vary substantially in how primary healthcare is organised and delivered [9]. For example, in the USA, providers specialising in women’s health are considered part of the primary care workforce. A recent review found that women’s health providers report feeling more comfortable with respect to both breast cancer risk assessment and primary prevention, resulting in greater reported use of risk assessment tools [12]. Therefore, there may be fewer challenges with implementing a breast cancer risk assessment and primary prevention pathway in primary care in the USA or other countries with different primary care systems.** | Pg 21 & 22, lines 477-485 |
| Detailed Methodology: The manuscript would benefit from a more detailed description of the thematic analysis process, including coding procedures and how themes were identified and validated.  Recommendation: The thematic analysis process should be described in more detail. | We have described the thematic analysis process in more detail as suggested. The text now reads:  Primary data analysis was conducted by the lead author with input from LG, JAU-S, and DPF. Initial coding was deductive to ensure the study objectives were addressed. Additional codes were also developed inductively during data analysis to capture nuances in the data**. The majority of coding was semantic, capturing explicitly expressed meaning and staying close to the language of participants. However, there was a shift towards more latent coding as the analysis progressed.** The lead author constructed a working thematic framework of codes contained within categories which was reviewed and refined as coding progressed by way of regular meetings with LG and JUS before being applied to all remaining transcripts. The ‘framework’ feature in NVivo12 was used to plot each category onto a separate thematic matrix with codes presented in separate columns, and participants (cases) on separate rows. Data in each cell were summarized to synthesise the data set and illustrative extracts were noted. The resulting data in the framework matrices were compared across and within cases to highlight similarities and differences and develop **initial** ~~the final~~ themes. Regular meetings were held between members of the research team (SH, LG, JAU-S, and DPF) throughout analysis and interpretation to discuss explanations for findings, **review and evaluate initial themes,** and **ultimately** determine the overall thematic structure. **The final thematic structure was agreed by the entire research team as representing participants’ views.** | Pg 8 & 9, lines 192-208 |
| Implementation Feasibility: While the manuscript discusses the challenges and preferred involvement of primary care providers, it could further elaborate on specific strategies or frameworks for integrating this pathway into existing primary care practices.  Recommendation: Suggest concrete frameworks or strategies for integrating the risk assessment and prevention pathway into primary care. | We have suggested the use of existing appointments as a potential vehicle for implementing risk assessment into primary care.  The text now reads:  Future research should focus on developing and evaluating strategies for implementing multifactorial risk assessment within primary care, taking into account **the need to minimise any additional workload, potentially through the use of additional allied healthcare professionals, the strong preference for governance of any pathway to sit outside individual primary care practices, and provision of adequate training, particularly around the prescription of Tamoxifen.** ~~the views of primary care, and paying particular attention to clinical governance requirements and how delivery can minimise exacerbation of health inequalities.~~ **Options include, for example, integrating risk assessment with consultations led by pharmacists, allied healthcare professionals or nurses related to prescription/review of contraception and cervical cancer screening. However, concerns have been raised about causing additional anxiety to these already anxiety-provoking appointments [21]. These approaches should be piloted to ascertain whether these concerns are well-founded.** **Furthermore,** b~~B~~etter modelling and communication of the numbers of women involved per general practice is needed to inform the scope of primary care’s involvement. | Pg 22, lines 491-502 |
| Literature Review: The introduction could provide a more comprehensive review of existing risk prediction models and their current use in primary care. | Thank you for this suggestion. A comprehensive review of existing risk prediction models is beyond the scope of the present paper, but we have added detail regarding the most commonly used models including the risk factors they assess. The text now reads:  The development of risk prediction models**, such as the Tyrer–Cuzick and BOADICEA models,** has made it possible to estimate an individual woman’s risk of developing breast cancer with reasonable levels of accuracy **[9, 10]**. **Each model differs on the ‘classic’ risk factors they incorporate into their risk estimates; this includes hormonal and reproductive risk factors, previous biopsies, and hereditary risk components (family history). More recently, risk prediction models have incorporated polygenic risk scores and mammographic breast density, which have improved their discriminatory accuracy** **[11, 12].**  References added:  9. Lee A, Mavaddat N, Wilcox AN, Cunningham AP, Carver T, Hartley S, et al. BOADICEA: a comprehensive breast cancer risk prediction model incorporating genetic and nongenetic risk factors. Genet Med. 2019;21(8):1708-18.  10. Tyrer J, Duffy SW, Cuzick J. A breast cancer prediction model incorporating familial and personal risk factors. Stat Med. 2004;23(7):1111-30.  11. Hurson AN, Pal Choudhury P, Gao C, Hüsing A, Eriksson M, Shi M, et al. Prospective evaluation of a breast-cancer risk model integrating classical risk factors and polygenic risk in 15 cohorts from six countries. Int J Epidemiol. 2021;50(6):1897-911.  12. Vilmun BM, Vejborg I, Lynge E, Lillholm M, Nielsen M, Nielsen MB, et al. Impact of adding breast density to breast cancer risk models: a systematic review. Eur J Radiol. 2020;127:109019. | Pg 3, line 62-68 |
|  | We have also expanded on the results of a recent systematic review which found that reported use of risk assessment tools in primary care is low. The text now reads:  A recent systematic review showed that primary care providers typically take a reactive role in breast cancer risk assessment that is predominantly focused on the collection of family history**, and report infrequent use of multi-factorial risk assessment tools.** | Pg 4, lines 77-78 |
| Clarity in Results: Some of the quotes from participants could be more clearly linked to the themes and subthemes to enhance the coherence of the results section.  Recommendation: Link participant quotes to the relevant themes and subthemes. | Thank you for this feedback. We have moved some quotes and amended the text in places so that the links between quotes and the narrative are clearer. Examples include:  Therefore, provision of a training and education package **to equip primary care providers with the knowledge needed to answer patient queries about the pathway** ~~address deficits in knowledge~~ was perceived as essential prior to involvement.  *I can just think of the spectrum of questions that women are going to be asking about their risk, about different contraceptive choices, IVF, you know. […] I can just imagine, a lot of the questions, we just might not have the answers to. (GP trainee, 6, FG2)*  Despite their concerns, ~~participants acknowledged that a change in practice was possible and~~ made suggestions about how primary care could be made to feel more comfortable assuming responsibility for managing Tamoxifen. For example, if a specialist in secondary care wrote the first prescription or a NICE guideline was developed focused on the initiation and ongoing management of Tamoxifen for breast cancer risk reduction in the target population. **Therefore, participants acknowledged that a change in practice was possible:**  I guess there are things that maybe ten, fifteen years ago that we would never have foreseen - that we wouldn’t have felt comfortable doing in primary care, that we now do. So, you know, things do change, and a lot of sometimes our beliefs are held because of what we’re traditionally used to doing. […] If you look at the management of diabetes, you know, that’s transformed in the last ten years and we’ve got used to doing it. (GP, 13, FG4) | See results section, pg 11-18 |
| Include more details under Ethical Considerations: Write clearer on how participant confidentiality was ensured. | We have added more detail to the procedure section describing how participant confidentiality was ensured. The text now reads:  Online focus groups and interviews were conducted between 6th July 2022 and 10th March 2023, using Zoom video conferencing. Following consent, participants gave demographic information (gender, age, ethnicity, employment details (job title and years in current role) and practice postcode). **For focus groups, participants provided this information to one of the facilitators, either via telephone or in a breakout room. Practice postcode was collected to assess the deprivation decile of the practice location using the Index of Multiple Deprivation 2019, a measure of relative deprivation for small areas in England [30]. Once the decile was extracted, the postcode was destroyed preserving the confidentiality of the participants.** Data were audio‐recorded and transcribed verbatim by an external transcription company. **Identifiable information was removed from the transcripts, and participants were assigned a study ID consisting of their profession and a number.** | Pg 8, lines 173-180 |
